# Is spike frequency adaptation an artefact? Insight from human studies

**DOI:** 10.3389/fncel.2012.00050

**Published:** 2012-10-31

**Authors:** Grzegorz Wilanowski, Maria Piotrkiewicz

**Affiliations:** Department of Engineering of Nervous and Muscular System, Nałęcz Institute of Biocybernetics and Biomedical Engineering, Polish Academy of SciencesWarsaw, Poland

## Introduction

Spike frequency adaptation (SFA) is defined as the decline in motoneuron (MN) firing rate during constant current injection. In classical study of Granit et al. (1963) this phenomenon was investigated in anesthetized animals through injecting MNs with long-lasting rectangular current steps. At least two phases of SFA have been observed in these experiments: the rapid initial phase and the slow (S) late phase (Kernell, 1965).

Historically, the first mechanism proposed to explain SFA was the summation of the medium afterhyperpolarization (Kernell, 1972; Kernell and Sjoholm, 1973; Baldissera et al., 1978). However, it has been shown that this mechanism may be responsible only for few initial MN interspike intervals (Powers et al., 1999). More recent studies of the possible mechanisms underlying late SFA have been usually conducted *in vitro* and often supported by computer simulations (e.g., Sawczuk et al., 1997; Zeng et al., 2005). In these experiments, a multitude of ion channels were blocked with appropriate pharmacological agents and the effects of blocking on SFA magnitude and/or time course were studied. With this type of protocol, certain mechanisms contributing to SFA were identified in non-MN cells. However, in MNs SFA appears to be such a robust phenomenon that blocking of any presumed mechanisms has no effect on its magnitude or time course. Thus, it was concluded that several membrane channels involved in generating rhythmic MN activity contribute to SFA. These mechanisms act together to ensure SFA stability: blocking one set of channels results in an increase in the contribution of the others (Powers et al., 1999; Zeng et al., 2005).

One piece of evidence supporting this theory of redundancy was presented by Goh et al. (1989) through experiments involving bullfrog sympathetic neurons. Under normal conditions, selective blockade of the delayed rectifier potassium current (I_K_) has no effect on SFA. However, SFA would be enhanced through blocking I_K_, when other I_K_s (including the calcium-dependent current I_AHP_) were blocked. Thus, the contribution of I_K_ to MN firing patterns depends on the activity of other K^+^ currents.

Considerable part of the research on SFA was produced by Brownstone's lab (Miles et al., 2005; Brownstone, 2006). Recently, they reported the reversal of SFA during fictive locomotion of decerebrate cat (Brownstone et al., 2011). During the recent meeting of International Motoneurone Community in Sydney the title question of this paper was posed (Brownstone, 2012). The author explains further his concern, asking: “… is repetitive firing produced by current injected through the micropipette … informative about membrane currents during behaviour? Perhaps SFA is not present … during most motor behaviours?” These questions reflect the author's conviction that a role for late SFA has not been established yet.

Human MN studies offer a possibility to investigate intact MNs in their physiological environment. In our opinion, these studies provided enough evidence to answer the questions cited above. This evidence will be presented below.

## Initial SFA

**Figure 1:**
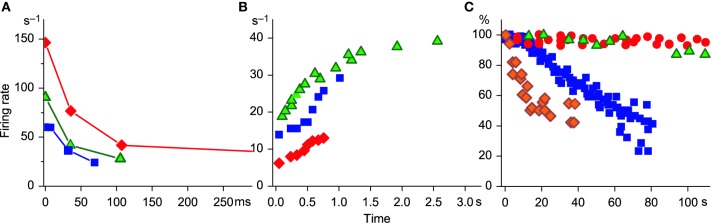
**SFA time course. (A)** Initial adaptation. Replotted data from Figure 6 of Granit et al. (1963): diamonds 10.2 nA, triangles, 5.7 nA, and Figure 4C (ballistic contraction) of Desmedt and Godaux (1977), squares. **(B)** Initial acceleration. Replotted data from Spielmann et al. (1993) (triangles, their Figure 3A) and from Desmedt and Godaux (1977), squares (Figure 4C, ramp contraction 12 kG/s); D2 (1.2 kG/s, Figure 4E); diamonds, unpublished data from a MN additionally recruited during sustained contraction. **(C)** Late adaptation. Squares, MN type F; circles, MN type S: replotted data from Spielmann et al. (1993) (their Figures 5A,B, respectively); diamonds, replotted data from Bigland-Ritchie et al. (1983a), (their Figure 4). Is kindly acknowledged; triangles, (Person and Kudina (1972), their Figure 3). The permission from Wiley and Sons and from Elsevier is kindly acknowledged. Note that the ordinate in **(A)** and **(B)** is scaled in cycles per second and in **(C)** in percent (data normalized by the initial value of firing rate); abscissa in **(A)** is scaled in milliseconds and in **(B)** and **(C)** in seconds.

The initial SFA phase is present only when the MN starts firing in response to the intracellular rectangular current injection. Since such a rapid depolarization seems to be absolutely non-physiological, this phase is most likely a candidate to be an artefact. However, there is an analogy of rectangular current step in human experiments, when the experimental subject is asked to contract the muscle as quickly as possible (so-called ballistic contractions). This task evokes a sudden increase in synaptic inflow to MN pool, resulting in recruitment of a few MNs with initial firing rates of 60–120 imp/s, which quickly decrease thereafter. The time course of this decrease is similar to those observed in animal MNs in response to constant current injection (Figure 1A).

The reaction of the MN to the rectangular current step was investigated by Ito and Oshima (1965). They showed that cat MNs respond to it with an abrupt change in membrane potential, which initially overshoots the target potential, peaks at approximately 15 ms after current onset and stabilizes around 100 ms. The magnitude of the overshoot increased with increasing current intensity. The time course of the initial SFA phase typically observed with rectangular current stimulation appears to follow changes in membrane voltage: the initial firing rate positively correlates with current step amplitude, and the steady level is reached at or shortly after 100 ms (Granit et al., 1963).

In contrast, during ramp contractions MN firing rate is gradually increasing until the contraction force reaches the target level (Desmedt and Godaux, 1977). Similar firing rate acceleration was observed in cat MNs, when the rectangular current step was applied extracellularly (Spielmann et al., 1993) and during sustained voluntary contractions in human MNs that were additionally recruited as muscle force gradually decreased (Person and Kudina, 1972; Piotrkiewicz et al., 1981) (see Figure 1B for comparison). The authors suggested that in this case the stimulation strength was progressively developing in the MN.

Yet another type of stimulation (intermediate between first two) was applied to cat MNs in the study of Baldissera et al. (1982), who investigated MN firing rate changes following “ramp and hold”-type current injections. The MN responded initially with a gradual increase in firing rate, followed by an overshoot with the peak occurring at approximately the time of transition between the ramp and the constant current, and stabilizing thereafter. The magnitude of this overshoot was positively correlated with the ramp slope and at the S ramps it virtually disappeared. It is conceivable that the transition from fast (F) ramp to steady current would generate a voltage overshoot analogous to that revealed by Ito and Oshima (1965).

## Late SFA

Late SFA develops in MN over several minutes and does not depend on the type of excitation, but on the type of muscle fibers that the MN innervates. MNs supplying F muscle fibers have significantly higher initial firing rates and faster late adaptation (Kernell and Monster, 1982; Spielmann et al., 1993) than those supplying S fibers. Firing rate slowing, comparable to this adaptation phase, has also been observed in human MNs during sustained voluntary contractions (Person and Kudina, 1972; Bigland-Ritchie et al., 1983a). In these experiments, the classification of MN to F and S subtypes was not possible. However, it is commonly known that F-type MNs are preferentially recorded during maximal voluntary contractions (MVCs) and those recorded at low force levels usually belong to S-type. During MVC, the firing rate decline was faster for MNs firing at higher rates (Bigland-Ritchie et al., 1983a). According to the recent study by Oya et al. (2009), these MNs also have higher recruitment thresholds. In Figure 1C, the SFA time courses for cat and human MNs are compared. It is obvious that the plot of a high-threshold human MN from the study of Bigland-Ritchie et al. (1983a) is comparable to the plot of cat MN type F, whereas the plot of a human MN recorded during submaximal contraction (Person and Kudina, 1972) appears similar to the plot of MN type S (Spielmann et al., 1993).

## The role of SFA

The reason that SFA in MNs is such a robust phenomenon is presumably related to the unique role of MNs as the Sherrington's “final common pathway” leading to muscle contraction. For MNs to play this role, their properties must be properly matched to those of their muscle units. This matching is indeed observed (Kernell et al., 1999).

As a result of this matching, MN firing rate is adjusted to the requirements of motor task performance. A short initial ISI, for example, is beneficial for smoothness and speed of F contractions (Baldissera and Parmiggiani, 1975; Stein and Parmiggiani, 1979). However, initial SFA is necessary for prolonging the ISI to adjust the firing rate to muscle contractile properties. During precise manipulations, MNs fire in the range close to the maximum slope of the force-rate dependency (Kernell et al., 1983) that corresponds to the maximum tetanic potentiation (Piotrkiewicz and Celichowski, 2007). During sustained MVCs, the MN firing rate should lead to full tetanus of its muscle unit. Although, a MN is able to fire with rates exceeding those necessary for achieving full tetanus, firing at excessive rates is not compatible with optimal contraction control (Bigland-Ritchie et al., 1983a) and may even be destructive to muscle fibers [in Duchenne muscular dystrophy, the pathological process is presumably enhanced by the mismatch between the muscle fiber properties and MN firing rates (Vrbova, 1983)]. Muscle units gradually change their contractile properties during fatigue and become slower, which means that the full tetanus is reached at the lower rates. Thus, the SFA-related decline in the firing rate allows the proper fit between muscle and MN properties to be preserved (Bigland-Ritchie et al., 1983a,b). This concept was denominated “muscle wisdom” by Marsden et al. (1971) and is accepted by the majority of researchers as the rationale for SFA (Spielmann et al., 1993; Zeng et al., 2005; Nordstrom et al., 2007).

Initially, human MN rate slowing during sustained contractions was thought to be mediated by a peripheral reflex from the fatiguing muscle (Bigland-Ritchie et al., 1986; Woods et al., 1987). This hypothesis, however, was later tested and ruled out (Butler et al., 2003; McNeil et al., 2011), which led to the conclusion that for rate slowing intrinsic MN properties were most likely responsible. The same conclusion was reached in the earlier study (Johnson et al., 2004) in which human subjects were instructed to maintain the steady firing rate of a given MN during prolonged contraction. During this task, excitatory input to the MN was substantially increased, indicating that intrinsic MN properties were underlying the observed rate slowing.

Moreover, the match between MN and muscle fiber properties is preserved in normal aging. The firing rate slowing is related to increases in MN AHP duration (Piotrkiewicz et al., 2007, 2012), which corresponds to changes in muscle contractile properties (Frontera et al., 1997).

SFA has usually been studied in animal experiments in response to long-lasting rectangular current steps. The physiological analogue of this artificial condition may occur in extreme situations when the animal or human being is falling from a dangerous height so that survival depends on the ability to quickly catch possible support and to hold on to it long enough for rescue. In this case, SFA would help economize necessary forces.

However, there are certain motor control tasks in which the firing rate decline is not desirable, such as in breathing or locomotion. Rhythmic movements may be continued without fatigue for periods lasting much longer than sustained contractions of constant force. In this case, neuromodulators may reverse SFA through metabotropic pathways. This reversal has been recently observed during fictive locomotion (Brownstone et al., 2011).

In summary, there is adequate evidence that SFA is not an artefact, but rather a manifestation of “neuromuscular wisdom,” which assures proper functioning of the healthy neuromuscular system under all physiologic circumstances.
